# Prenatal and postnatal diagnosis of Phelan–McDermid syndrome: A report of 21 cases from a medical center and review of the literature

**DOI:** 10.3389/fgene.2022.961196

**Published:** 2022-08-31

**Authors:** Ying Hao, Yang Liu, Jingxin Yang, Xingping Li, Fuwei Luo, Qian Geng, Suli Li, Peining Li, Weiqing Wu, Jiansheng Xie

**Affiliations:** ^1^ Medical Genetic Center, Affiliated Shenzhen Maternity & Child Healthcare Hospital, Southern Medical University, Shenzhen, Guangdong, China; ^2^ Department of Genetics, Yale University School of Medicine, New Haven, CT, United States; ^3^ Reproductive Medicine and Prenatal Diagnosis Centre, Division of Prenatal Diagnosis, The University of Hong Kong-Shenzhen Hospital, Shenzhen, Guangdong, China

**Keywords:** Phelan–McDermid syndrome (PMS), 22q13.3 deletion syndrome, SHANK3 gene, prenatal diagnosis, genotype–phenotype correlations

## Abstract

**Background:** Phelan–McDermid syndrome (PMS), caused by deletions at 22q13.3 and pathogenic variants in the *SHANK3* gene, is a rare developmental disorder characterized by hypotonia, developmental delay (DD), intellectual disability (ID), autism spectrum disorder (ASD), dysmorphic features, absence of or delayed language, and other features.

**Methods:** Conventional karyotyping, chromosomal microarray analysis (CMA), and whole exome sequencing (WES) have been used to detect genetic defects causing PMS. We summarized the genetic and clinical findings from prenatal to postnatal stages of detected cases of PMS and mapped potential candidate haploinsufficient genes for deletions of 22q13. This study aimed to summarize the laboratory findings, genetic defects, and genotype–phenotype correlations for Chinese patients with PMS.

**Results:** Seven prenatal cases and fourteen postnatal cases were diagnosed with PMS in our center. Thirteen cases had a deletion ranging in size from 69 to 9.06 Mb at 22q13.2-q13.33, and five cases had a pathogenic variant or an intragenic deletion in the *SHANK3* gene. Three familial cases with a parental carrier of a balanced translocation were noted. A review of the literature noted another case series of 29 cases and a report of five cases of PMS in China. Genotype–phenotype correlations confirmed haploinsufficiency of the *SHANK3* gene for PMS and suggested other candidate haploinsufficient genes *TNFRSFI3C* and *NFAM1* genes for immunological features and *TCF20, SULT4A1*, *PARVB, SCO2*, and *UPK3A* genes for intellectual impairment and behavioral abnormality, neurological features, macrocephaly/hypotonia, oculopathy, and renal adysplasia, respectively.

**Conclusion:** Indications for prenatal diagnosis of PMS are not specific, and approximately 85% prenatally diagnosed PMS elected termination of pregnancies after genetic counseling. For postnatal cases, 62.5% were caused by a deletion at 22q13 and 37.5% were caused by a pathogenic variant or an intragenic deletion in the *SHANK3* gene. Approximately 6.7% of cases with a deletion were familial, and almost all pathogenic variants were *de novo*. Combined karyotype, CMA, and WES should be performed to increase the diagnostic yield. The identification of other candidate haploinsufficient genes in deletions of 22q13.2-q13.33 could relate to more severe dysmorphic features, neurologic defects, and immune deficiency. These results provided evidence for diagnostic interpretation, genetic counseling, and clinical management for the Chinese cases of PMS.

## Introduction

Phelan–McDermid syndrome (PMS, OMIM #606232) is a rare developmental disorder with diverse clinical features. It was first described in 1985 (Watt et al., 1985) and later defined as 22q13.3 deletion syndrome characterized by neonatal hypotonia, absent to severely delayed speech, developmental delay (DD), autism spectrum disorders (ASDs), moderate to profound intellectual disability (ID), minor dysmorphic facial features, and behavioral characteristics including mouthing or chewing non-food items and decreased perception of pain (Phelan and McDermid., 2012).

The prevalence of PMS is unknown, but to date, the PMS Foundation had registered more than 2,800 individuals (https://www.pmsf.org/, accessed 1 Jun 2022). PMS is mainly caused by a heterozygous deletion at chromosome 22q13.3 involving the *SHANK3* gene or a pathogenic variant in the gene (Sarasua et al., 2014). The *SHANK3* gene plays a critical role in synaptic function and dendrite formation by encoding a scaffolding protein enriched in the postsynaptic density of glutamatergic synapses (Kolevzon et al., 2014). Deletions or pathogenic variants in the *SHANK3* gene have been identified in patients ascertained for ASD and ID with an estimated frequency at 0.5–2%, respectively (Kolevzon et al., 2014).

Two case series including 34 cases of 22q13 deletions and *SHANK3* pathogenic variants have been reported in China (Gong et al., 2012; Xu et al., 2020). Here, we reported 21 previously undescribed Chinese cases with PMS from our medical center. The findings from these three case series revealed the types of genetic defects and the spectrum of clinical findings from prenatal to postnatal stages. These results provide guidance for effective laboratory diagnosis on affected patients and evidence for proper genetic counseling on follow-up parental study and clinical management.

## Materials and methods

### Case series

This retrospective study was approved by the Medical Ethical Committee of Shenzhen Maternity and Child Healthcare Hospital (SZMCH), and informed consent for prenatal testing was obtained from pregnant women. From 2015 to 2021, 12,280 of prenatal cases and 940 of pediatrics cases were subjected for genetic analysis in SZMCH and 21 cases (seven prenatal and 14 postnatal) were diagnosed as PMS. Parents or guardians were contacted for genetic counseling using a standardized questionnaire of medical records and family history and follow-up genetic testing. The questionnaire included questions about the developmental, neurological, behavioral, and additional clinical features in the affected children and other relatives. Ten families also provided pictures of their affected children. A review of the literature found a report of five cases in China and another case series of 29 Chinese cases (Gong et al., 2012; Xu et al., 2020). Genetic defects and clinical findings from prenatal to postnatal stages were summarized and compared for these reported cases and our case series.

### Genetic analysis

Conventional G-banded karyotyping was performed on peripheral blood or amniotic fluid specimens using standardized protocols. A CMA was performed using a single-nucleotide polymorphism (SNP) array platform by Affymetrix CytoScan 750K arrays (Thermo Fisher Scientific Inc., Waltham, Massachusetts, United States) following the manufacturer’s protocols. WES was performed by KingMed Diagnostics, Running Gene, Baylor Miraca Genetics Laboratory, and our center. All detected pathogenic variants were further confirmed by Sanger sequencing at our center using a ABI3500Dx Genetic Analyzer (Applied Biosystems, Foster City, CA).

### Statistical analysis

Collectively, 48 postnatal cases with clinical findings were summarized from our cases and the other two case series. These patients were divided into two groups to evaluate the correlated phenotypes. Group I (*n* = 30) included individuals with deletions that encompass the *SHANK3* gene. Group II (*n* = 18) were individuals with a pathogenic variant or an intragenic deletion in the *SHANK3* gene. Fisher’s exact test was used to assess statistical significance in important phenotypes between the two groups. The results were judged to be statistically significant at *p* < 0.05.

## Results

From 2015–2021, genetic evaluation has been performed for a total of 12,280 prenatal and 940 postnatal cases in SZMCH. Seven prenatal cases and 14 postnatal cases were diagnosed with PMS, indicating a detection rate of 0.06% and 1.5% in the prenatal and postnatal cases, respectively, and a detection rate of 0.16% of the total cases. By karyotyping, CMA, and follow-up WES and sanger sequencing, 16 cases (76%) were detected with a deletion at 22q13.3 and three of them were familial cases resulted from a parental carrier of a balanced translocation. Five cases (24%) had a pathogenic variant or an intragenic deletion in the *SHANK3* gene.

### Cytogenomic characterization of deletions of 22q13.3

**FIGURE 1:**
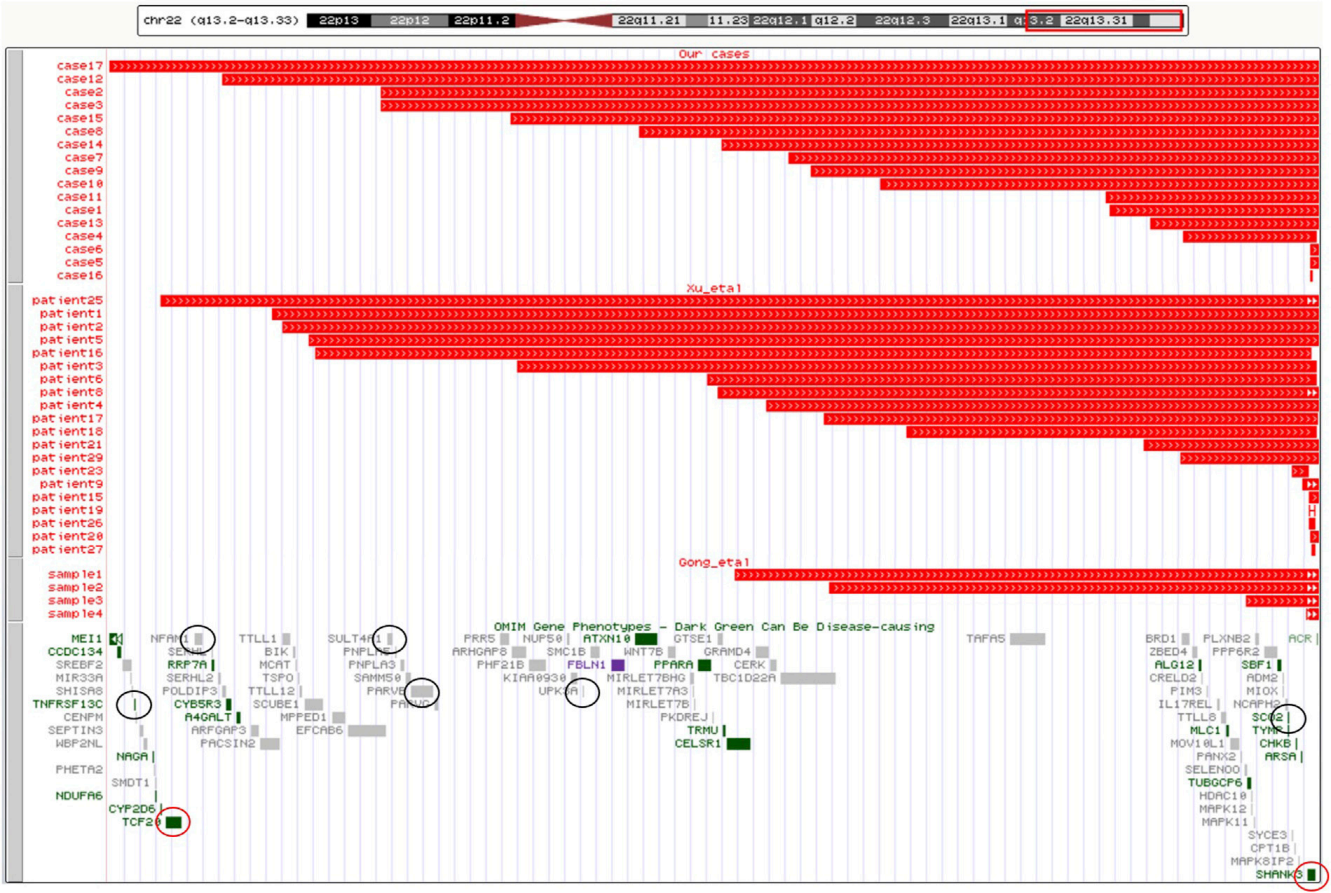
Cytogenomic mapping and genotype–phenotype correlations for candidate genes of 22q13.2-q13.33. A cytogenomic map for sizes of deletions and candidate genes of 22q13.2-q13.33. The upper panel shows chromosome 22 with the band location information. The region of 22q13.2-q13.33 is boxed in red. The middle panel shows the size and location of the 41 deletions: 17 cases in our study, 20patients in the literature of Xu et al. (2020), and four samples in the literature of Gong et al. (2012). The lower panel shows OMIM genes in 22q13.2-q13.33. The HI genes are circled in red and six other genes associated with the PMS phenotype are circled in black.

The 16 cases with detected cytogenomic abnormalities are summarized in Table 1. All 16 cases carried a deletion ranging from 69 Kb to 9.06 Mb at 22q13.2-q13.33 (Figure 1).

**TABLE 1:** Details of the 22q13.3 deletions identified in 16 individuals with PMS.

Case^a^	Age (years)	Karyotype	CMA results [hg19 (GRCh37)]	Deletion or duplication (size, kb)	Number of OMIM genes	Inheritance
1	Fetus	46,XN	arr 22q13.33 (49628163_51197766)x1	1,570	29	*De novo*
2	Fetus	46,XN,del (22) (q13.2)	arr 22q13.2q13.33 (44164976_51197766)x1	7,033	56	*De novo*
3	4	46,XY,del (22) (q13.2)	arr 22q13.2q13.33 (44164976_51197766)x1	7,033	56	*De novo*
4	13	46,XX,der (22)t (21;22) (q22.2;q13.3)mat	arr 21q22.3 (42548815_48068066)x3	5,519	67	Mat
			arr 22q13.33 (50172717_51178405)x1	1,006	30	
5	Fetus	46,XN	arr 22q13.33 (51128325_51197766)x1	69	2	*De novo* ^b^
6	Fetus	46,XN	arr 22q13.33 (51128324_51197766)x1	69	2	*De novo*
7	Fetus	46,XN,der (22)t (15;22) (p11.2;q13.31)pat	arr 22q13.31q13.33 (47220048_51197766)x1	3,987	31	Pat
8	Fetus	46,XN,del (22) (q13)	arr 22q13.31q13.33 (46102178_51197766)x1	5,096	43	NA
9	2	NA	arr 22q13.31q13.33 (47383286_51197766)x1	3,814	31	*De novo*
10	13	NA	arr 22q13.31q13.33 (47909900_51197766)x1	3,288	30	NA
11	11	NA	arr 22q13.33 (49596615_51197766)x1	1,601	29	*De novo*
12	10	46,XX,del (22) (q13)	arr 22q13.2q13.33 (42977177_51197766)x1	8,221	67	*De novo*
13	9	46,XY,der (22)t(8;22) (q24.3;q13.33)mat	arr 8q24.3 (140110416_146295771)x3	6,185	72	Mat
			arr 22q13.33 (49928521_51197766)x1	1,269	29	
14	6	46,XY	arr 22q13.31q13.33 (46718620_51197766)x1	4,479	36	NA
15	8	46,XX,del (22) (q13)	arr 22q13.31q13.33 (45143535_51197766)x1	6,054	49	NA
16	Fetus	46,XN,del (22) (q13)	arr 22q13.2q13.33 (42135216_51197766)x1	9,063	84	*De novo*

a Seven prenatal cases: 1, 2, 5, 6, 7, 8, and 16; nine postnatal cases: 3, 4, 9–15. Three familial cases: 4, 7, and 13.

b Parental genetic testing by qPCR, while the others by CMA.

NA, not available; CMA, chromosomal microarray analysis; OMIM, Online Mendelian Inheritance in Man; pat, paternal; mat, maternal.

**FIGURE 2:**
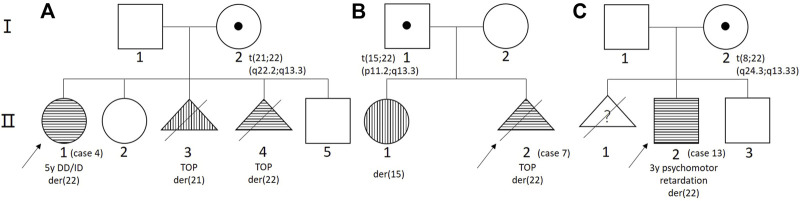
The pedigree of three cases from three families. **(A)** Family 1; **(B)** family 2; and **(C)** family 3.

Parental genetic testing was performed on 12 cases, of which nine cases had a *de novo* deletion and three cases from three families had a derivative chromosome from a parent carrying a balanced translocation. In family 1 (Figure 2A), the mother had a balanced translocation t (21;22) (q22.2;q13.3) and the proband (case 4/II-1 in pedigree) inherited a derivative chromosome 22 with a terminal duplication of chromosome 21q22.3. This proband was diagnosed at the age of 5 years because of DD and ID. Follow-up genetic analysis on the mother’s four consecutive pregnancies detected two pregnancies with a derivative chromosome 21 or 22 (II-3 and II-4) and two pregnancies with a normal karyotype (II-2 and II-5). After genetic counseling, the parents elected the termination of pregnancy (TOP) for the two with abnormal cytogenomic findings. In family 2 (Figure 2B), the proband (case 7/II-2) had a derivative chromosome 22 from the father carrying a balanced translocation t (15;22) (p11.2;q13.3). A previous pregnancy had a girl (II-1) carrying a derivative chromosome 15 who had mild cleft lip and normal intelligence. The parents elected the TOP after genetic counseling. In family 3 (Figure 2C), the first pregnancy of the woman was induced by premature rupture of membranes at 24 weeks of gestation without genomic test. The proband (case 13/II-2) was evaluated at the age of 3-years for psychomotor retardation. He carried a derivative chromosome 22 with a terminal duplication of 8q24.3 and a deletion of 22q13.33. His mother’s karyotype was 46,XX,t (8;22) (q24.1;q13.3). The couple then had a boy with completely normal chromosomes through preimplantation genetic diagnosis (PGD).

### Pathogenic and likely pathogenic variants in the *SHANK3* gene

The five cases with an intragenic deletion and pathogenic and likely pathogenic variants are summarized in Table 2. Case 17 was detected with a *de novo* intragenic deletion of 13.66 Kb involving exons 10 to 17 of the *SHANK3* gene, and this deletion will disrupt the transcription and translation. Four cases (18–21) were detected with a pathogenic or likely pathogenic variant in the *SHANK3* gene by WES. Since Kolevzon et al. (2011) reported that the human genome reference assembly (GRCh37/hg19) misses the beginning of exon 11, we corrected the position of nucleotides and amino acids according to the *SHANK3* mRNA (NM_001372044.2) and protein (NP_001358973.1) in RefSeq, respectively . The variants included three frameshift variants and one nonsense variant and all of them were *de novo*.

**TABLE 2:** Intragenic deletion and four pathogenic variants in the *SHANK3* gene of five cases.

Case	Age (years)/gender	Variant location^a^	Protein change^b^	Position (hg19)	Location	Variant type	Inheritance	ACMG classification
17	12/M	Intragenic del 13.66 kb	Disruption	g.51132803_51146462	Exon 10–17	Del	*De novo*	Pathogenic
18	5/M	c.3730del	p.Arg1244GlyfsTer13	g.51159805	Exon 21	Frameshift	*De novo*	Pathogenic
19	4/F	c.4093C>T	p.Arg1365Ter	g.51160168	Exon 21	Nonsense	*De novo*	Pathogenic
20	11/F	c.3288del	p.Ile1097SerfsTer43	g.51159363	Exon 21	Frameshift	*De novo*	Likely pathogenic
21	13/M	c.3938_3950 del	p.Ala1313ArgfsTer29	g.51160013_51160025	Exon 21	Frameshift	*De novo*	Pathogenic

a NM_001372044.2.

b NP_001358973.1.

M, male; F, female; ACMG, American College of Medical Genetics.

### Clinical findings in prenatal cases

A total of seven pregnancies (cases 1, 2, 5, 6, 7, 8, and 16) were detected prenatally with a deletion of 22q13.2-q13.33, and the TOP was elected in six pregnancies. The pregnant woman of case 2 chose to give birth to a fetus with PMS. Pregnancies for cases 9, 11, 12, and 14 had clinical indications for prenatal genetic evaluation but did not select CMA, and postnatal follow-up analysis by CMA detected a deletion of 22q13.2-q13.33. For case 18, prenatal karyotyping and CMA tests by the concern of X-ray exposure found a normal result and follow-up WES at the age of 5 years detected a pathogenic variant in the *SHANK3* gene.

The indications for prenatal analysis included parental carriers (case 7), nuchal translucence (cases 6 and 7), increased risk of Down syndrome by maternal serum screening (cases 1 and 5), advanced maternal age (case 5), abnormal finding in the noninvasive prenatal testing (NIPT) (case 1), and fetal anomalies by ultrasound examination. Fetal growth restriction, single umbilical artery, complex congenital heart disease, jejunal atresia, cystic dysplasia of the left kidney, obstructive reflux renal injury, and persistent left superior vena cava was each observed on one fetus. Multiple structural abnormalities were found in case 16, including morphological changes of skull, local premature closure of coronal suture, short long bones, early ossification of distal femur epiphyseal cartilage, abnormal rib shape, enlarged cardiothoracic ratio, polycystic dysplasia of the right kidney, and the dermal medulla of the left kidney is unclear.

Thirteen cases of PMS diagnosed prenatally in the literature were reviewed to compare the clinical indications for analysis, gestational age at diagnosis, methods, results, inheritance, and pregnancy outcomes (Table 3). In addition to the prenatal diagnostic indications mentioned in our study, additional fetal abnormalities were observed, including ventricular septal defect, choroid plexus cyst, total anomalous pulmonary venous return, ventricle prominence, truncus arteriosus, right microtia, hemivertebrae of the lumbar spine, unilateral multicystic kidney, unilateral cleft lip both left-sided, fetal ascites, and ventriculomegaly. 13 prenatal cases were combined with our seven cases, CMA and karyotype should be performed to detect the genomic imbalance in the chromosome structural rearrangements, and 85% (17/20) of pregnancies elected the TOP after genetic counseling.

**TABLE 3:** Clinical features in pregnancies of PMS fetuses.

Literature	Gestational age at prenatal diagnosis (wks)	Methods	Reason for prenatal diagnosis	Results	Inheritance	Pregnancy outcomes
Shen et al. (2020)	The second trimester	CMA	Ventricular septal defect with choroid cyst of the right ventricle	arr 22q13.31q13.33 (46009655-51197766)x1, 21q21.1q21.2 (20088087-24600640)x1	*De novo*	TOP
Ge et al. (2020)	23	CMA	NIPT shows microduplication of chromosome 22	arr 22q13.2q13.33 (44088529-51197766)x1	*De novo*	TOP
Ge et al. (2020)	22	CMA	Increased risk of Down’s syndrome by maternal serum screening	arr 22q13.31q13.33 (44620384-51197766)x1, 22q11.1q13.2 (16888899-42985310)x2-3	*De novo*	Continued gestation
Luo et al. (2019)	25	CMA	NIPT shows microduplication of chromosome 11	arr 22q13.31q13.33 (47167879-51197838)x1	*De novo*	TOP
Chen et al. (2016)	28	CMA	Right microtia, hemivertebrae of the lumbar spine, ventricular septal defect, and total anomalous pulmonary venous return	arr 22q13.31q13.33 (44731454-51209196)x1	*De novo*	TOP
Kirkpatrick and El-Khechen, (2011)	24	Karyotype, FISH	Unilateral multicystic kidney and unilateral cleft lip both left-sided, polyhydramnios	46,XX,del (22) (q13.1)dn.ish del (22) (q13.31) (ARSA-,22qSUBTEL-)	*De novo*	Live birth
Chen et al. (2010)	20	CMA	Fetal ascites and ventriculomegaly	Partial trisomy 16p (16p12.2-pter), partial monosomy 22q (22q13.31-qter)	Parental carriers	TOP
Koç et al. (2009)	17	Karyotype, FISH	Abnormal finding in the maternal serum screening test, unilateral small choroid plexus cyst	mosaic ring 22 dup/del with terminal 22q13 del	*De novo*	TOP
Maitz et al. (2008)	21	Karyotype, FISH	Complex congenital heart defect, a significant right ventricle prominence	46,XX,ish del (22) (q13.3q13.3) (ARSA-,D22S1726+)	NA	TOP
Delcán et al. (2004)	16	Karyotype, FISH	NA	46,XX, r22	*De novo*	TOP
Chen et al. (2003)	18	Karyotype	Advanced maternal age, intrauterine growth restriction, a ventricular septal defect, and truncus arteriosus	45,XX,-22 [6]/46,XX,r (22) (p13q13.31)[15]	*De novo*	TOP
Phelan et al. (2001)	17	Karyotype, FISH	Increased risk of Down syndrome (1/57)	46,XX,del (22) (q13.3)[19]/46,XX [1]	*De novo*	TOP
Riegel et al. (2000)	21	Karyotype, FISH	Cystic tumor in the fetal neck and the upper thoracic aperture	Mosaicism for a distal 22q deletion in fetal fibroblasts	*De novo*	TOP

TOP, termination of pregnancy; CMA, chromosome microarray analysis; FISH, fluorescence *in situ* hybridization; NIPT: noninvasive prenatal testing.

### Clinical findings in postnatal cases

**FIGURE 3:**
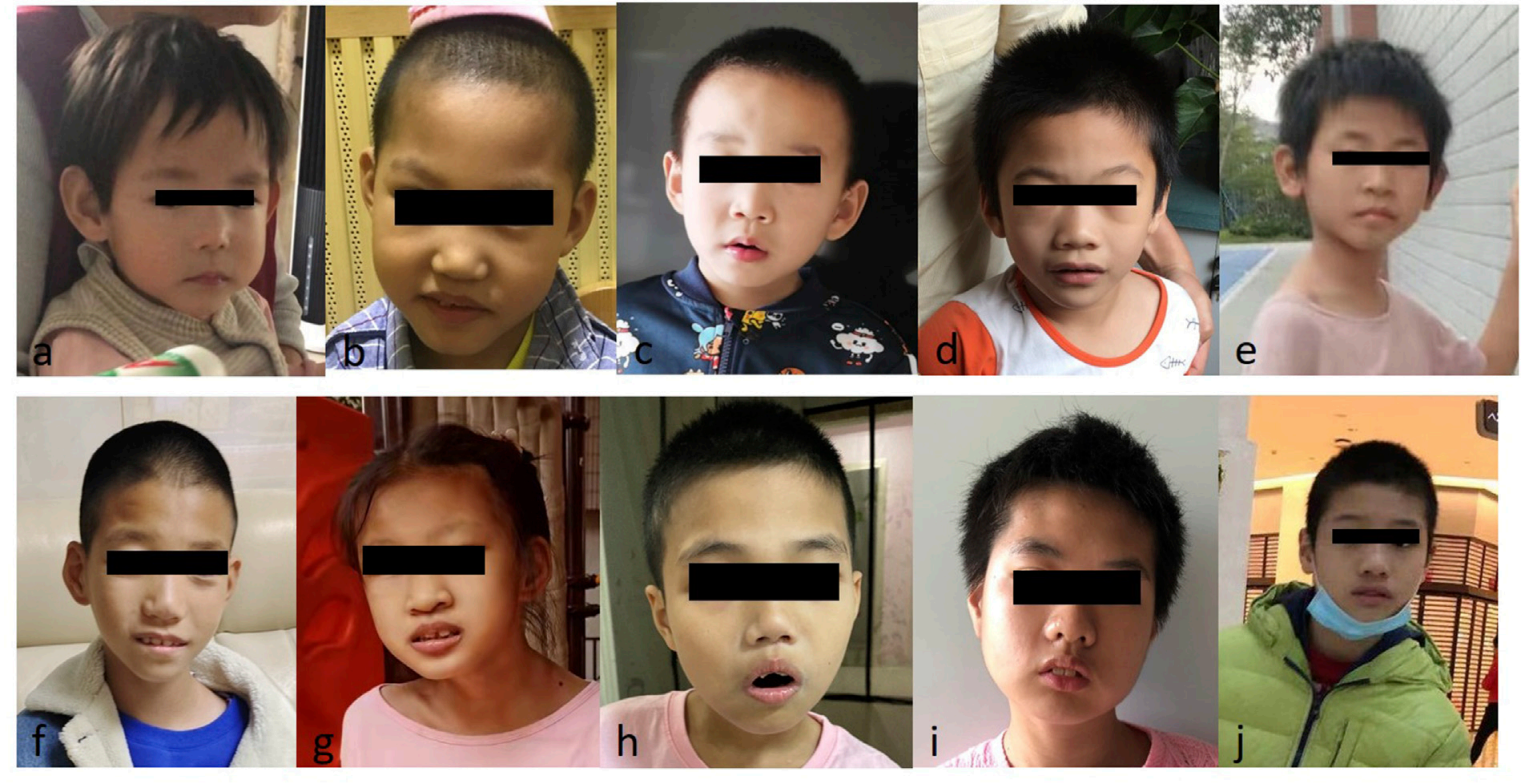
Images of patients with PMS. **(A)** Case 9, 2 years old; **(B)** case 3, 4 years old; **(C)** case 14, 6 years old; **(D)** case 16, 7 years old; **(E)** case 15, 8 years old; **(F)** case 13, 9 years old; **(G)** case 12, 10 years old; **(H)** case 20, 11 years old; **(I)** case 10, 13 years old; and **(J)** case 21, 13 years old. **(H,J)** Patients with pathogenic variants of the *SHANK* gene; others: the patients with 22q13 deletion encompass the *SHANK3* gene or an intragenic deletion of this gene.

As shown in Figure 3, patients with PMS had mild to moderate facial dysmorphism. There was no significant difference between group I patients with 22q13 deletions encompassing the *SHANK3* gene and group II patients with a pathogenic variant or an intragenic deletion in the *SHANK3* gene.

Of the 48 postnatal cases from our case series and two other reports, there were 26 boys and 21 girls with an average age of 6 years (except for one case whose sex and age were unknown). 30 cases (63%) were group I with a deletion at 22q13 and 18 cases (37%) were group II with a pathogenic variant or an intragenic deletion in the *SHANK3* gene. Of the 11 cases of group I with follow-up familial study, 82% of the deletions at 22q13 were *de novo*, 18% were derived from maternal carriers of a balanced translocation. 13 cases of group II with follow-up parental analysis showed normal results in both parents indicating *de novo* variants in the patients. The clinical features observed from our study and two case series are summarized in Table 4. The frequency of diarrhea/constipation in patients of group II was higher than that in group I (50% vs. 17%) and the difference was statistically significant (*p* = 0.041). There was no significant difference between group I and group II in the frequency of clinical features including hypotonia (72% vs. 94%), ASD (60% vs. 92%), and repetitive behaviors (65% vs. 71%), which are higher in group II, absent or severely delayed speech (age ≥ 3) (76% vs. 62%), decreased perception of pain (71% vs. 67%), and gait abnormalities (72% vs. 53%) are higher in group I, while DD/ID (93% vs. 94%) and hyperactivity (83%) are almost equal in both groups.

**TABLE 4:** Comparison of postnatal phenotypes between patients with deletions at 22q13 and pathogenic variants or an intragenic deletion in the *SHANK3* gene than *SHANK3* gene and loss of *SHANK3* alone.

Clinical features	Group I: 22q13 deletions encompass the *SHANK3* gene			Total	Group II: pathogenic variants or an intragenic deletion in the *SHANK3* gene			Total	*p* value
	Current study	Xu et al. (2020)	Gong et al. (2012)		Current study	Xu et al. (2020)	Gong et al. (2012)		
Developmental/neurological									
DD/ID	9/9 (100%)	13/15 (87%)	4/4 (100%)	26/28 (93%)	5/5 (100%)	10/11 (91%)	1/1 (100%)	16/17 (94%)	1
Absent or severely delayed speech (age ≥ 3)	8/9 (89%)	8/12 (67%)	3/4 (75%)	19/25 (76%)	5/5 (100%)	3/8 (38%)	NA	8/13 (62%)	0.457
Seizures (febrile and/or non-febrile)	4/6 (67%)	4/17 (24%)	NA	8/23 (35%)	1/3 (33%)	3/12 (25%)	NA	4/15 (27%)	0.728
Decreased perspiration/tendency to overheat	2/6 (33%)	4/17 (24%)	NA	6/23 (26%)	1/2 (50%)	2/12 (17%)	NA	3/14 (21%)	1
Decreased perception of pain	6/7 (86%)	11/17 (65%)	NA	17/24 (71%)	3/3 (100%)	7/12 (58%)	NA	10/15 (67%)	1
Arachnoid cyst	1/7 (14%)	3/17 (18%)	NA	4/24 (17%)	0/3 (0%)	1/12 (8%)	NA	1/15 (7%)	0.631
Hypotonia	8/8 (100%)	12/17 (71%)	1/4 (25%)	21/29 (72%)	4/5 (80%)	12/12 (100%)	NA	16/17 (94%)	0.124
Gait abnormalities	8/8 (100%)	10/17 (59%)	NA	18/25 (72%)	2/3 (67%)	6/12 (50%)	NA	8/15 (53%)	0.31
Brain imaging abnormalities	2/7 (29%)	9/12 (75%)	1/2 (50%)	12/21 (57%)	1/3 (33%)	4/9 (44%)	NA	5/12 (42%)	0.481
Behavioral features									
Autism spectrum disorder (age ≥ 3)	4/6 (67%)	10/17 (59%)	2/4 (50%)	6/10 (60%)	4/4 (100%)	7/8 (88%)	NA	11/12 (92%)	0.135
Chewing difficulties/mouthing/tooth grinding	7/8 (88%)	5/17 (29%)	NA	12/25 (48%)	2/4 (50%)	5/12 (42%)	NA	7/16 (44%)	1
Biting (self or others)	5/5 (100%)	5/17 (29%)	NA	10/22 (45%)	3/3 (100%)	4/12 (33%)	NA	7/15 (47%)	1
Nonstop crying (crying nonstop for 3 h)	1/5 (20%)	6/17 (35%)	NA	7/22 (32%)	0/3 (0%)	7/12 (58%)	NA	7/15 (47%)	0.493
Hyperactivity	6/7 (86%)	14/17 (82%)	NA	20/24 (83%)	0/0 (0%)	10/12 (83%)	NA	10/12 (83%)	1
Pica	3/7 (43%)	1/17 (6%)	NA	4/24 (17%)	0/2 (0%)	2/12 (17%)	NA	2/14 (14%)	1
Repetitive behaviors	4/6 (67%)	11/17 (65%)	NA	15/23 (65%)	1/2 (50%)	9/12 (75%)	NA	10/14 (71%)	0.735
Sleep disturbance	3/8 (38%)	4/17 (19%)	NA	7/25 (28%)	2/2 (100%)	3/12 (25%)	NA	5/14 (36%)	0.723
Other clinical features									
Gastroesophageal reflux	3/7 (43%)	2/17 (12%)	NA	5/24 (21%)	0/2 (0%)	0/12 (0%)	NA	0/14 (0%)	0.137
Diarrhea/constipation	1/6 (17%)	3/17 (24%)	NA	4/23 (17%)	4/4 (100%)	4/12 (33%)	NA	8/16 (50%)	0.041
Eczema	0/7 (0%)	4/17 (24%)	NA	4/24 (17%)	1/3 (33%)	4/12 (33%)	NA	5/15 (33%)	0.266
Recurring upper respiratory tract infections	6/8 (75%)	5/17 (29%)	NA	11/25 (44%)	1/2 (50%)	1/12 (8%)	NA	2/14 (14%)	0.083
Cardiac abnormalities	2/7 (29%)	2/17 (12%)	NA	4/24 (17%)	0/3 (0%)	1/12 (8%)	NA	1/15 (7%)	0.631
Allergies	1/7 (14%)	3/17 (24%)	NA	4/24 (17%)	2/3 (67%)	2/12 (17%)	NA	4/15 (27%)	0.686
Asthma	0/7 (0%)	1/17 (7%)	NA	1/24 (4%)	1/3 (33%)	0/12 (0%)	NA	1/15 (7%)	1
Renal abnormalities	2/6 (33%)	3/14 (21%)	NA	5/20 (25%)	1/3 (33%)	0/8 (0%)	NA	1/11 (9%)	0.383

NA, not available; DD, developmental delay; ID, intellectual disability. The cases of 22q13 deletions encompass *SHANK3* gene in the current study, Xu et al. (2020), and Gong et al. (2012) are 9, 17, and 4, respectively. The cases of pathogenic variants or an intragenic deletion in the *SHANK3* gene in the current study, Xu et al. (2020) and Gong et al. (2012) are 5, 12, and 1, respectively. The fraction in the table whose denominator is less than these numbers are the cases with loss to follow-up for this phenotype.

### Genotype–phenotype correlations within deletions of 22q13.2-q13.33

Possible phenotypic correlations were evaluated based on the gene content within the deletions of 22q13.2-q13.33. There were 41 cases of PMS detected with a deletion of 22q13.2-q13.33, including 17 from our cases and 24 from other two case series. The largest deletion spans about 9 Mb. After excluding pseudogenes, there are 102 coding genes in the 22q13.2-q13.33 region. Of them, 24 OMIM morbid genes, two haploinsufficient genes, *TCF20* and *SHANK3* (by ClinGen HI score = 3), and 13 putative haploinsufficient genes, *SCUBE1*, *SULT4A1*, *PHF21B*, *FBLN1*, *CELSR1*, *GRAMD4*, *BRD1*, *PIM3*, *PLXNB2*, *SBF1*, *MAPK8IP2*, *TSPO,* and *PPARA* (by ClinGen pLI ≥ 0.90 or %HI ≤ 10%), were listed as candidate genes. The cytogenomic map for sizes of deletions and candidate genes of 22q13.2-q13.33 are shown in Figure 1. Of all the 41 cases, deletions of case 16 and patient 25 fully contained the *TCF20* and *SHANK3* genes, deletions of 28 cases contained the *SHANK3* gene completely, seven cases (case 5 and 6, sample 4, patient 15, 16, 20, and 23) contain a partial deletion of the *SHANK3* gene and both breakpoints of deletion in four cases (case 17, patient 19, 26, and 27) are within the *SHANK3* gene.

## Discussion

In a recent large cohort of 210 PMS individuals, the different genomic rearrangements were distributed as follows: 90% (189/210) of deletions, 6.9% (13/189) of familial cases by parental carriers, and 10% (21/210) of pathogenic variants (Nevado et al., 2022). In Chinese patients, it was 62.5% (30/48) of deletions, 6.7% (2/30) of familial cases by parental carriers, and 37.5% (18/48) of pathogenic variants. Comparing the two groups showed that the Chinese patients had a lower percentage of deletions and higher percentage of pathogenic variants. A collection of more Chinese patients is needed to define that this difference represents a true ethnic variation or technically under detection of deletions at 22q13.3 by CMA.

The detection rate of deletions at 22q13.3 for PMS is 0.12% (16/13,220) in our prenatal and postnatal cases and is detected in 0.06% of pediatric cases in a report. The prevalence of PMS caused by deletions is estimated as 1/12,500 in the Chinese population (Chai et al., 2019).

It is estimated that 15–20% of PMS are resulted from an unbalanced chromosome rearrangement involving chromosome 22, of which approximately 50% are inherited from a balanced carrier parent (Phelan and McDermid, 2012). In our case series, the detection rate of parental carriers of a balanced translocation was 19% (3/16), which is consistent with that reported in the literature. In family 2, the phenotype of group II was mild compared to the classical features of PMS, suggesting a weak triplosensitive effect for the duplication of this region. In family 3, case 13 carried additional CNV of 8q24.3 duplication, which can cause profound psychomotor retardation, idiopathic epilepsy, and growth delay (Bonaglia et al., 2005) similar to PMS. This is the first familial case of a balanced translocation showing compound effects from a distal duplication of 8q24.3 and a deletion of 22q13.33 in the Chinese population. Approximately 20% of Chinese PMS caused by chromosomal rearrangements were familial cases, and parental genetic testing should be performed to provide complete genetic information for genetic counseling and clinical management.

For the prenatal cases of PMS, the clinical indications for prenatal diagnosis varied. There are no specific prenatal indications for PMS. Most fetal abnormalities observed by ultrasound were fetal growth restriction and abnormalities of the heart, kidney, or bone. Enhanced noninvasive prenatal testing (NIPT) could screen for chromosomal deletions despite a certain percentage of false positive and false negative results. When chromosomal abnormalities were suggested by NIPT, invasive prenatal diagnosis is necessary to provide a diagnosis. CMA should be recommended as the method of choice to detect chromosomal deletions and duplications.

Comparisons of phenotypes between the two groups of 48 cases in China showed that there was no significant difference in the clinical features including DD/ID, absence of speech, and ASD in accordance with a previous study (Xu et al., 2020). We also demonstrated that there was no significant difference (*p* = 0.124) in the clinical feature of hypotonia with a high frequency in group II than group I (94% vs. 72%). However, hypotonia frequency was slightly higher in the loss of *SHANK3* gene alone group and it has statistical significance near the borderline in their study (*p* = 0.059). Interestingly, Levy et al. (2021) found early infancy hypotonia and hypotonia persisting after infancy were higher in group I deletions (deletions encompassing more than *SHANK3* gene) than in group II intragenic deletions (include *SHANK3* only or several nearby genes) with statistically significant *p-*values (*p* = 0.001 and *p* = 0.006). Larger deletion size has also been correlated with greater severity and presence of numerous clinical features, including hypotonia (Luciani et al., 2003; Wilson et al., 2003; Sarasua et al., 2011). These differences may be related to limited sample size, ethnics, some phenotypes that had not yet appeared causing the low mean age, and limitation from obtaining medical history (through medical record review or questionnaires completed by parents instead of assessing the patients by professional physician). The larger sample sizes and more detailed studies are needed for further clinical presentations.

As 22q13.2-q13.33 is a rich gene region, many genes might be responsible for neurodevelopmental features. There are three haploinsufficient genes *SHANK3*, *TCF20,* and *EP300* in this region. The *SHANK3* gene is at 22q13.3 and the other two genes are located in 22q13.2. According to the ClinGen Dosage Sensitivity, these three genes all have a HI score and a pLI score which is intolerant of LoF (HI = 3, pLI = 1). Loss of function due to haploinsufficiency of the *SHANK3* gene can lead to PMS and it has been well-documented.

*TCF20* (OMIM 603107), which encodes the protein transcription factor 20 contains over 1,900 amino acids and harbors several functional domains. It is predicted to function as a transcriptional activator or repressor depending on its interaction with other factors (Gburcik et al., 2005). It is highly expressed in the brain and strongly linked to severe neurodevelopmental phenotypes, encompassing cognitive and motor deficits, autistic traits, attention deficit hyperactivity disorder (ADHD), craniofacial dysmorphisms, macrocephaly, body overgrowth, muscle hypotonia, seizures, constipation, scoliosis, strabismus, myopia, and keratoconus (Torti et al., 2009). Cases (case 17 and patient 25) with large deletion containing both the *SHANK3* and *TCF20* genes have a more severe phenotype in our study. The deletion of 22q13.2 not containing the *SHANK3* gene showed that the *TCF20* gene is a candidate gene for the neurodevelopmental features (Upadia et al., 2018). Similar interstitial microdeletions were found in two patients presenting with classic clinical features of PMS in the 22q13.2 region that map proximal to the *SHANK3* gene (0.54 and 0.72 Mb, respectively) (Simenson et al., 2014; Thümmler et al., 2016). It is worth noting that duplication including the *TCF20* gene was suspected to cause a neurodevelopmental disorder (NDD) with mirror traits compared to patients with *TCF20* deletions (Lévy et al., 2021). The *TCF20* gene is essential for neurogenesis and is ubiquitously expressed in the developing cerebral cortex, notably in the neural stem cells of mouse embryos (Feng et al., 2020). In particular, *TCF20* knockout mice lead to neurogenesis defects with imbalanced differentiation and proliferation of neural progenitor cells and lead to autistic-like behaviors in mice. The abnormal regulation of neurogenesis usually leads to NDDs comprising ASD, ADHD, and ID. The *TCF20* gene is essential for neurogenesis and the author suggest that *TCF20* duplication could also lead to defects in neurogenesis (Lévy rt al., 2021).

*The EP300* gene is not involved in 41 cases of PMS in China (Figure 1). Pathogenic variants and deletions of the *EP300* gene are implicated in Rubinstein–Taybi syndrome. Larger duplications including the *EP300* gene could be associated with a more severe phenotype with growth delay and global DD (Feng et al., 2020). *EP300* has also been implicated as a tumor suppressor gene (Samanich et al., 2012).

*TNFRSF13C*, an OMIM Morbid gene in this region, shows that LoF mutations are a known cause of primary immunodeficiency. *NFAM1* is another gene that is involved in B-cell development and signaling. As previously mentioned (Simenson et al., 2014; Thümmler et al., 2016), both patients presented with developmental delay, language delay, and physical features of bilateral ptosis, bulbous nasal tip, and clinodactyly. Moreover, they also had immunological features of elevated immunoglobulin E (Simenson et al., 2014), repetitive bronchiolitis, and persistent asthma (Thümmler et al., 2016). Both microdeletions involve the *TNFRSFI3C* and *NFAM1* genes, which are involved in immune system functioning and are postulated as candidate genes for the immunological features seen in these patients.

Disciglio et al. (2014) reported nine cases of interstitial 22q13 deletions not involving the *SHANK3* gene. In the minimal deleted region, they identified two candidate genes, *SULT4A1* and *PARVB* (associated with the PTEN pathway), which could be associated with neurological features and macrocephaly/hypotonia, respectively. Its high expression in specific regions of the brain suggests a role for *SULT4A1* in the central nervous system and has been implicated in schizophrenia. They suggest that the deletion of this gene may be associated with neurological symptoms in these patients. The haploinsufficiency of *PARVB* gene may be responsible for hypotonia by affecting the colocalization of dysferlin at the sarcolemma of skeletal muscle.

Another two genes with an autosomal dominant mechanism are *SCO2* and *UPK3A*, whose evidence of dominant effects is less consistent. *SCO2* plays a critical role in cytochrome c oxidase (COX or complex IV) function in mitochondria (Frye et al., 2016). Pathogenic mutations can produce a severe form of autosomal dominant myopia (Tran-Viet et al., 2013). *UPK3A* is a component of the uroplakins family, expressed on the transitional epithelium covering the urinary tract. *De novo* heterozygous missense variants were found associated with renal adysplasia or hypodysplasia (Jenkins et al., 2005).

Different clinical presentations observed among PMS patients carrying identical deletions and the surprising degree of severity displayed by some patients carrying very small deletions clearly pointed toward the modifying effects played by other genetic and environmental factors. These genetic factors may include additional small indels, variants, or allelic variation in still unidentified genes as well as epigenetic modification and noncoding RNAs with an active role in transcriptional regulation (Ricciardello et al., 2021).

Treatment of PMS potentially encompassing dozens of genes and disrupting regulatory elements altering gene expression and inferring the potential for multiple therapeutic targets. At present, there is no single best treatment for PMS. Approaches to therapy are necessarily complex and must target variable behavioral and physical symptoms of PMS.

## Conclusion

This is the first detailed report of the prenatal and postnatal diagnosis of PMS at a single Chinese medical center. The detection rate of PMS in the prenatal and postnatal diagnosis is 0.06% and 1.5%, respectively. We summarized the clinical findings from prenatal to postnatal stages of PMS in China. The indications for prenatal diagnosis of PMS are not specific and there is a need of enhanced NIPT to screen for chromosomal deletions. There is no significant difference in major phenotypes between cases of 22q13 deletions encompassing the *SHANK3* gene and cases with a pathogenic variant or an intragenic deletion in the *SHANK3* gene. The analysis of genotype–phenotype correlations for deletions of 22q13.2-q13.33 indicated that the haploinfficiency of the *SHANK3* is sufficient to cause PMS and deletion extended to the *TCF20* gene could lead to more severe neurologic defects. The *TNFRSFI3C* and *NFAM1* genes are postulated as candidate genes for the immunological features seen in these patients. The *SULT4A1*, *PARVB, SCO2,* and *UPK3A* genes are associated with neurological features, macrocephaly/hypotonia, oculopathy, and renal adysplasia, respectively. The haploinsufficiency and synergy of these genes introduce variable clinical features for patients of PMS. More clinical cases and further functional research are needed to establish precisive genotype–phenotype correlations for better understanding of the complexity and variability of PMS.

## Data Availability

The datasets used and analyzed during the current study are included in Tables 1–4 and Figures 1–3. Detailed clinical findings on patients will be available by contacting the corresponding author.
